# Contemporary Management of Cardiac Implantable Electronic Device Infection: The American College of Cardiology COGNITO Survey

**DOI:** 10.1016/j.jacadv.2023.100773

**Published:** 2023-12-20

**Authors:** Dhanunjaya R. Lakkireddy, Archana Rao, Paul Theriot, Douglas Darden, Naga Venkata K. Pothineni, Rashmi Ram, Yu-Rong Gao, Jim W. Cheung, Ulrika Birgersdotter-Green

**Affiliations:** a Kansas City Heart Rhythm Institute, HCA Midwest, Overland Park, Kansas, USA; b Department of Cardiology, Liverpool Heart and Chest Hospital, Liverpool, United Kingdom; c Enterprise Content & Digital Strategy Division, American College of Cardiology, Washington, DC, USA; d Image Guided Therapy, Philips North American, Colorado Springs, Colorado, USA; e Division of Cardiology, Department of Medicine, Weill Cornell Medicine, New York City, New York, USA; f Division of Cardiology, University of California-San Diego Health System, San Diego, California, USA

**Keywords:** cardiac implantable electronic devices, extraction, guidelines, infection, pocket infection

## Abstract

**Background:**

Cardiac implantable electronic devices (CIEDs) infection remains a serious complication, causing increased morbidity and mortality. Early recognition and escalation to definitive therapy including extraction of the infected device often pose challenges.

**Objectives:**

The purpose of this study was to assess U.S.-based physicians current practices in diagnosing and managing CIED infections and explore potential extraction barriers.

**Methods:**

An observational survey was performed by the American College of Cardiology including U.S. physicians managing CIEDs from February to March 2022. Sampling techniques and screener questions determined eligibility. The survey featured questions on knowledge and experience with CIED infection patients and case scenarios.

**Results:**

Of 387 physicians completing the survey (20% response rate), 49% indicated familiarity with current guidelines regarding CIED infection. Electrophysiologists (EPs) (91%) were more familiar with these guidelines, compared to non-EP cardiologists (29%) and primary care physicians (23%). Only 30% of physicians specified that their institution had guideline-based protocols in place for managing patients with CIED infection. When presented with pocket infection cases, approximately 89% of EPs and 50% of non-EP cardiologists would follow guideline recommendation to do complete CIED system removal, while 70% of primary care physicians did not recommend guideline-directed treatment.

**Conclusions:**

There are gaps in familiarity of guidelines as well as the knowledge in practical management of CIED infection with non-extracting physicians. Most institutions lack a definite pathway. Addressing discrepancies, including guideline education and streamlining care or referral pathways, will be a key factor to bridging the gap and improving CIED infection patient outcomes.

Cardiac implantable electronic devices (CIEDs) play an important role in contemporary management of cardiac arrhythmias and reduce the risk of sudden cardiac death. Clinical indications for CIEDs have expanded, leading to a gradual yet significant increase in implantation rates over the past 2 decades.^1^ This rise in CIED implantation rates has also led to a parallel increase in CIED infection rates.^2^ CIED infection can be a serious and life-threatening condition which is associated with significant morbidity, mortality, and health care burden.^3^^,^^4^

Although CIED infections are a resource-intensive clinical burden, they are often underdiagnosed and associated with significant heterogeneity in clinical management. Inexperience with CIED infection recognition and management could increase the likelihood of missed infection, resulting in delayed and suboptimal management with poor outcomes. Therefore, adequate knowledge and skills regarding CIED infection management are needed among health care providers involved in the clinical care pathway to ensure timely identification and optimal management. Multiple clinical practice guidelines and consensus documents on prevention and management of CIED infection have endorsed Class I indications for complete system and lead removal in the presence of CIED infection.^5^, ^6^, ^7^, ^8^ However, major gaps in knowledge and insufficient adherence to guidelines continue to remain an important hurdle to overcome. A recent analysis of Centers for Medicare & Medicaid Services data demonstrated that more than 8 in 10 patients are not treated according to Class I guidelines.^9^ A comparable pattern is seen in a separate, extensive nationwide database of 25,000+ patients with CIEDs systemic infection revealed that only a small proportion of patients (12%) underwent transvenous lead extraction.^10^ This evidence underscores the critical need for a more in-depth exploration of the potential barriers to extraction and the gaps that currently exist in the timely management of CIED infection.

The COGNITO (COGNITO: Latin for awareness of Knowledge; ContempOrary manaGemeNt of cardiac ImplanTable electrOnic device infection) study was a survey conducted by the American College of Cardiology (ACC) to gain insights from the U.S. fellows of ACC members. The purpose of the survey was to capture and understand clinical perspectives related to the challenges in care and overall awareness of CIED infection.

## Methods

The ACC conducted an online survey designed to understand and assess the current practice of U.S.-based electrophysiologists (EPs), cardiologists (non-EPs), and primary care physicians (PCPs) related to the diagnosis and management of CIED infections from February 11, 2022, to March 10, 2022. The survey was based on a previous survey undertaken by European Heart Rhythm Association (EHRA) to explore the gaps in knowledge around CIED management and modified to suit the American health care system.^11^

The 10- to 15-minute questionnaire is provided in the Supplemental Appendix. The demographic section had 8 questions, and the main survey section included 17 questions. The survey was anonymized with no identifiable personal or patient data so specific ethical approval was not required. Participants were first asked to identify as an EP, non-EP cardiologist, or PCP, and also type of practice (hospital, university, multispecialty/physician group). Participants were also asked to select multiple-choice responses to the following: years of experience managing CIED, experience with device implantations and extractions, estimates of annual device infection at respective centers, familiarity with device extraction guidelines, whether institution has guidelines or protocols in place for the management of CIED infections, along with several management questions. Furthermore, 3 patient case scenarios involving CIED infections were provided with multiple questions.

### Recruitment

Four different sample sources were used when fielding the survey. Email invitations and 3 reminders were sent to ACC CardioSurve panelists. The CardioSurve Panel consists of 492 U.S.-based fellows of the ACC who have voluntarily agreed to participate in monthly research surveys for a 2-year term. Panelists were selected using a stratified random sampling technique to ensure accurate demographic representation of the U.S. cardiologists. For the EP audience, email invitations and 2 reminders were sent to 933 EP members of the ACC. For the PCP audience, email invitations and 2 reminders were sent to 500 PCP panel members of the Dynata research group. Twitter was also used as a channel for survey distribution. The survey was distributed on this channel via the ACC X handle (@ACCIntouch) and survey sponsor accounts. Three patient case scenarios were provided in the survey to assess competency regarding identification and management of CIED infections.

### Statistical analysis

The results are presented as absolute numbers as reported by the respondents. All the variables were categorical and expressed as percentages. The percentages were not adjusted for missing values. *P* values were calculated using chi-squared and Fisher exact tests. Statistical test comparisons were performed by participant type (ie, EPs, non-EPs, and PCPs) and also by years of CIED patient experience (ie, <7 years, 8-21 years, and 22 years or more). Specific group comparisons and corresponding *P* values are cited in the Results. Analysis of responses was performed using SPSS version 27.

## Results

### Demographics

A total of 387 physicians (80% male) completed the survey with an overall response rate of 20%. Of those who completed the survey, 190 were from CardioSurve (response rate 39%), 117 from EP ACC database (response rate 13%), 75 from Dynata database (response rate 15%), and 5 physicians from Twitter.

The distribution of the physicians was 134 (35%) EPs, 178 (46%) non-EP cardiologists, and 75 (19%) PCPs as shown in Table 1. Years in practice included 1% for <1 year, 18% for 1 to 7 years, 41% for 8 to 21 years, and 41% for 22 or more years. Primary practice setting of the participants was cardiovascular practice (32%), university (26%), hospital (14%), multispecialty group (14%), and physician group (13%). There was a bimodal distribution of practice location with 48% in urban areas and 40% in the suburbs. Respondents were equally spread over the East (29%), South (28%), West (22%), and North (21%) regions of the United States.

**Table 1:** Study Group Demographics and Results

	Specialty			
	EP (n = 134, 35%)	Cardiologist (Non-EP) (n = 178, 46%)	PCP (n = 75, 19%)	Total (N = 387)
Demographics				
Sex				
Male	85%	80%	72%	80%
Female	15%	20%	28%	20%
Years in practice				
<1 y	1%	1%	0%	1%
1-7 y	14%	27%	4%	18%
8-14 y	27%	21%	19%	23%
15-21 y	25%	11%	20%	18%
22 or more years	34%	39%	57%	41%
Primary practice setting				
CV practice	40%	39%	0%	32%
Medical school/university	30%	32%	4%	26%
Hospital	22%	13%	4%	14%
Multispecialty group	7%	15%	24%	14%
Physician group	0%	0%	65%	13%
HMO/industry	0%	0%	1%	<1%
Other	0%	1%	0%	<1%
Decline to answer	1%	0%	1%	1%
Number of cardiologists in practice				
Large (26 or more)	45%	44%	13%	38%
Medium (11-25)	27%	24%	8%	22%
Medium small (5-10)	16%	17%	13%	16%
Small (1-4)	9%	13%	23%	13%
None	1%	0%	36%	7%
No answer	2%	2%	7%	3%
Practice location				
Rural	7%	14%	9%	11%
Suburban	37%	33%	61%	40%
Urban	54%	52%	28%	48%
No answer	1%	1%	1%	1%
Experience				
Implant CIED, pacemaker, defibrillator	98%	9%	11%	40%
Perform lead extraction	46%	2%	11%	19%
Years of experience managing CIED patients				
None, no experience	0%	34%	61%	27%
1-7 years of experience	11%	25%	11%	17%
8-21 years of experience	55%	17%	21%	31%
22 or more years of experience	34%	24%	5%	24%

Values are %.

CIED = cardiac implantable electronic device; CV = cardiovascular; EP = electrophysiologist; HMO = health maintenance organization; PCP = primary care physician.

### Experience

Nearly three-fourths of respondents (73%) had some level of experience managing patients with CIEDs with EPs significantly more likely to treat CIED patients (100% EP, 66% non-EP cardiologists, 37% PCP; *P* < 0.001). Almost all of the EPs (98%) had implanted CIEDs (pacemaker, defibrillator, etc), while only 9% of non-EP cardiologists implanted CIEDs (*P* < 0.001). Clinicians who were more experienced with managing CIED patients also were more likely to implant these devices (8-21 years of CIED patient experience, 71%; 22+ years of CIED patient experience, 56%; *P* < 0.001). Lead extraction was performed by 19% of respondents, specifically 46% of the EPs. The 8 (11%) PCPs who performed CIED implantation and lead extraction indicated that in addition to being a PCP they were either hospitalists or internists.

### CIED infection management

**Figure 1:**
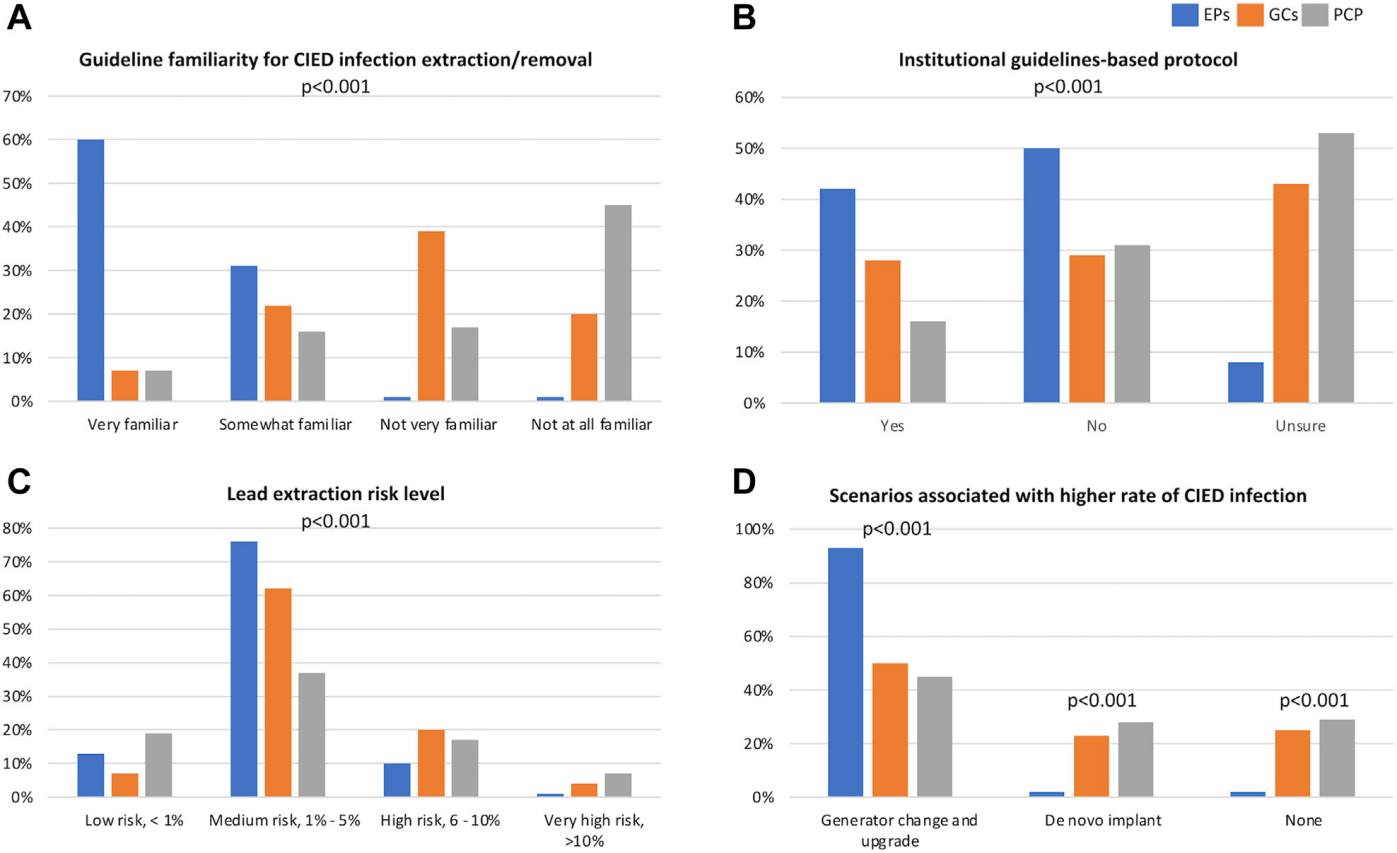
**Familiarity With Guidelines, Institution for Protocols, and Perception of CIED Infection Risk** (A) Familiarity with the current practice guideline recommendations for CIED infection extraction/removal. Most electrophysiologists (91%) were familiar with the guidelines. There was a significant difference among the 3 specialties in guideline familiarity (*P* < 0.001, chi-squared test). (B) Clinicians were divided on whether their institution/department has guidelines-based protocols for managing patients with CIED infections. There was a significant difference among the 3 specialties in their institutional guideline-based protocol for managing patients with CIED infection (*P* < 0.001, chi-squared test). (C) Physician perceived risk of lead extraction. Majority of physicians believed that lead extraction is a medium risk procedure. There was a significant difference among the 3 specialties in perceived risk of lead extraction procedure (*P* < 0.001, chi-squared test). (D) Physicians’ answers to the question regarding scenarios associated with higher rate of CIED infection. A high proportion of EPs believed that generator change and upgrade is a scenario that is typically associated with a high rate of CIED infection. There was a significant difference among the 3 specialties in their understandings of scenarios associated with CIED infections (multi-response question, *P* < 0.001 for every answer, chi-squared test).

EPs reported highest familiarity (91%) with the current practice guideline recommendations for CIED infection extraction/removal as compared to non-EP cardiologists (29%) and PCPs (23%) (*P* < 0.001) as shown in Figure 1. More than half of EPs (61%) estimated that the annual infection rate at their facility was <1% per year, as compared to 44% of non-EP cardiologists and 24% of PCPs. However, 60% of PCPs and 40% of non-EP cardiologists reported that they were unsure of the infection rate or it was not applicable to their facility. Fewer than one-third (30%) of respondents were aware of guidelines-based CIED infection protocols at their facility (EP: 42%, non-EP cardiologist: 28%, PCP: 16%; *P* < 0.001).

Three-fifths of clinicians (62%) reported that the risk level of major complications resulting from a lead extraction procedure was moderate (1%-5% risk), while 20% indicated that it was high risk (6%-10% risk) or very high risk (>10% risk). EPs (76%) and cardiologists (non-EPs) (62%) were more likely to report that the risk of major complications was moderate as compared to 37% of PCPs (*P* < 0.001). Almost all EPs (93%) associated a higher rate of CIED infection with the generator change and upgrade scenario as compared to non-EP cardiologists (50%) and PCPs (45%) (*P* < 0.001).

The top 2 decision factors to consider lead extraction or referral for extraction were the patient’s comorbidity (87%) and risk of lead extraction procedure (86%), followed by the age of the lead (80%) and the age of the patient (79%). Ease of access to extraction center had a much lower level of influence (55%). The least influential factor is the fear of losing the patient to the extraction physician (11%), although 25% of PCPs surveyed felt that this was an influence.

After diagnosing a patient with CIED pocket infection, 69% of respondents would refer to a device specialist with expertise in lead management, including 31% of EPs, 89% non-EPs, and 89% PCPs as shown in Table 2. Less than one-half (44%) would refer to an infectious disease specialist. Almost three-fourths of EPs who perform lead extraction (73%) would manage the patient on their own as compared to 39% of EPs who do not perform lead extractions. Approximately 22% would manage the patient on their own, including 54% EPs, 3% non-EPs, and 11% PCPs. After diagnosing a patient with a CIED with bacteremia, 61% would refer to an infectious disease specialist and 58% would refer to a device specialist with expertise in lead management.

**Table 2:** Management and Referral of Patients With Device Infection

	EP	Cardiologist (Non-EP)	PCP	*P* Value
Influences on decision to consider extraction or referral				
Patient’s comorbidity	95%	84%	77%	<0.001
Risk of lead extraction procedure	94%	86%	73%	<0.001
Age of the lead	90%	78%	67%	<0.001
Age of the patient	92%	74%	71%	<0.001
Ease of access to extraction center	44%	56%	73%	<0.001
Fear of losing patient to extraction physician if I refer	7%	7%	25%	<0.001
Action after diagnosing patient with CIED pocket infection				
Refer to device specialist with expertise in CIED management	31%	89%	89%	<0.001
Refer to infectious disease specialist	46%	45%	36%	0.32
Manage patient on own	54%	3%	11%	<0.001
Action after recognizing bacteremia in patient with CIED				
Refer to infectious disease specialist	59%	66%	55%	0.205
Refer to device specialist with expertise in CIED management	25%	77%	72%	<0.001
Manage patient on own	46%	8%	8%	<0.001
Often consult infectious disease specialist about CIED patient	81%	75%	47%	<0.001

Values are %.

CIED = cardiac implantable electronic device; EP = electrophysiologist; PCP = primary care physician.

### Patient case scenarios

**Figure 2:**
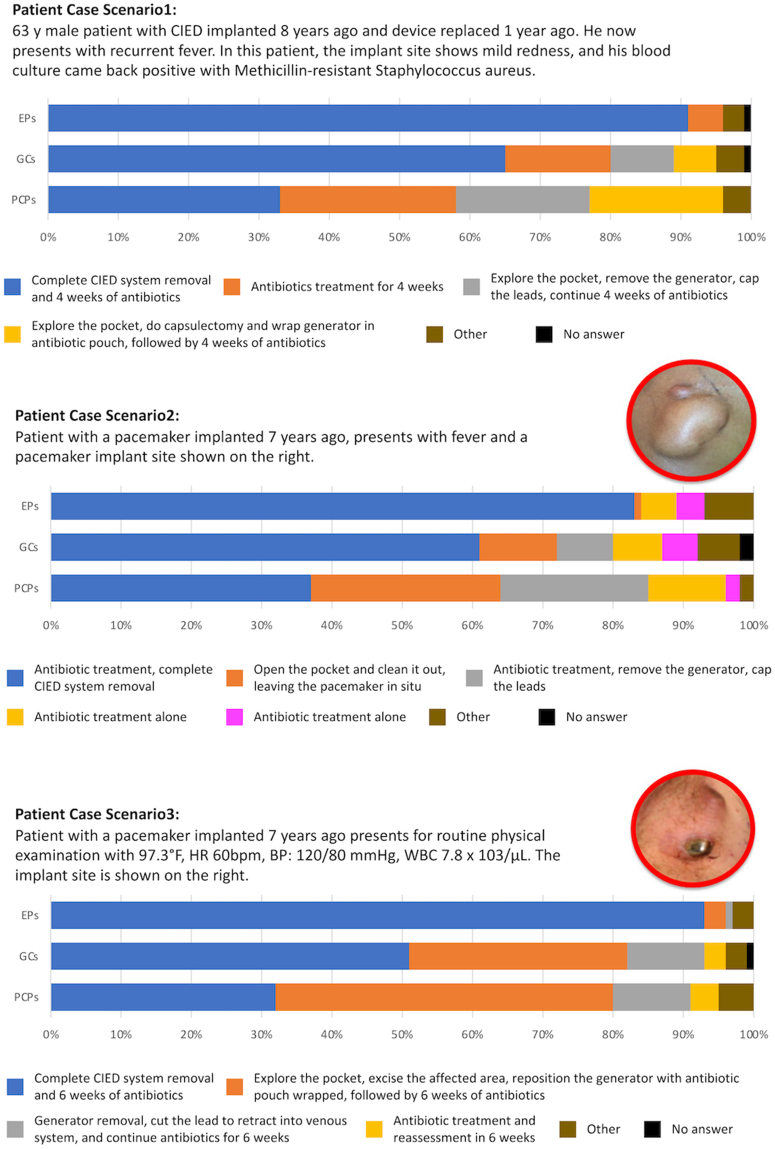
**Physician Responses to 3Case Scenarios**

Case scenarios are provided in the Figure 2. In case 1, a 63-year-old male with a CIED implanted 8 years ago presented with recurrent fever and found to have a pocket infection and methicillin-resistant *Staphylococcus aureus*. Most EPs (91%) reported that a complete CIED system removal and 4 weeks of antibiotics were the optimal therapy for this patient, as compared only 65% non-EPs and 33% PCPs (*P* < 0.001). Guidelines recommend that for a patient with suspected CIED pocket infection and positive blood cultures of *S aureus*, complete CIED system should be removed, and IV antibiotics should be administered for 4 to 6 weeks depending on the transesophageal echocardiogram findings. CIED should be reimplanted when blood cultures are negative for at least 72 hours and CIED remains indicated.

In case 2, a patient with a pacemaker implanted 7 years prior presented with a fever and pocket site swelling and erythema. Most EPs (83%), while only 61% of non-EPs and 37% of PCPs reported antibiotic treatment and complete CIED system removal as the next step (*P* < 0.001). Guidelines recommend that for a patient with signs of systemic infection, blood cultures should be ordered to determine the type of bacteria and transesophageal echocardiogram should be performed to examine valve vegetation. Complete CIED system should be removed, and antibiotics should be administered for 4 to 6 weeks. When blood cultures are negative for at least 72 hours and CIED remains indicated, reimplant CIED.

In case 3, a patient presented with a pacemaker pocket erosion. Patient was afebrile with normal white blood cell count. Most EPs (93%) reported that the next step for this patient is complete CIED system removal and 6 weeks of antibiotics, compared to 51% of non-EP cardiologists and 32% of PCPs (*P* < 0.001). Guidelines suggest that erosion of any part of the CIED should imply contamination of the entire system, complete system removal should be performed. Patients should also be treated with 7 to 10 days of antibiotics before reimplantation of a new CIED with specific timing dependent on clinical scenario and if CIED remains indicated.

## Discussion

The COGNITO study is the largest professional society-based survey of practicing physicians in assessing approaches to management of CIED infection. The survey represents a broad range of practicing clinicians including EPs, non-EP cardiologists, and PCPs. The main findings of the survey are as follows. First, familiarity with identification and management of CIED infections was very low among non-EP cardiologists and PCPs, as shown in the Central Illustration. Second, fewer than one-third of respondents had knowledge of local institutional CIED management protocols. Even among EPs who routinely implant CIEDs, only 42% reported being aware of institutional CIED infection protocols. Third, for a patient with CIED infection, approximately 40% of EPs who do not perform lead extraction reported managing the patient themselves without a referral to a lead extraction specialist. Additionally, 11% of PCPs reported managing CIED infections themselves. One-fourth (24%) of PCPs and one-fourth (24%) of non-EP cardiologists perceived lead extraction as a procedure with high (6%-10%) or very high (>10%) risk of major complications. Finally, perceived risk of lead extraction was a major determinant of referral practices in 86% of respondents.

**Central Illustration:**
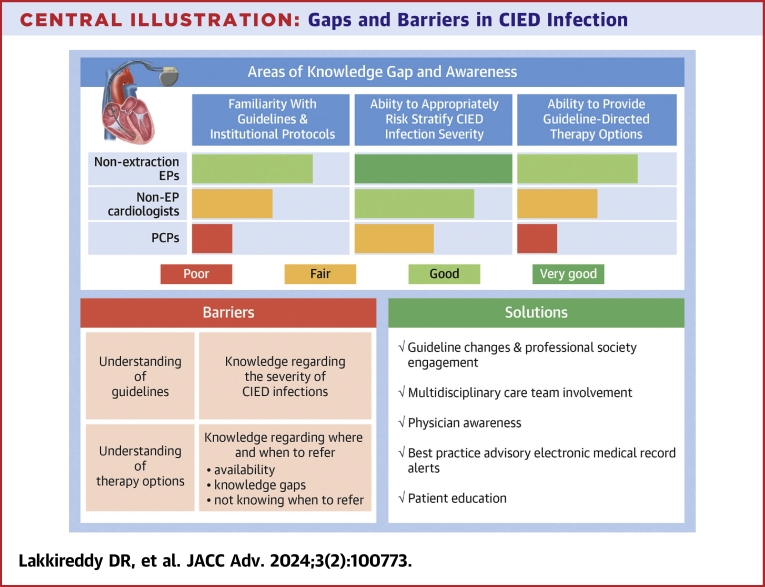
**Gaps and Barriers in CIED Infection** CIED = cardiac implantable electronic device; EMR = electronic medical record; EP = electrophysiologist; PCP = primary care physician.

A 2020 EHRA survey identified substantial gaps in the knowledge and skills of European physicians across all phases of CIED care. Comparing to the EHRA survey, our study shifts focus to U.S. physician groups to identify knowledge and skill gaps in an effort to understand the educational needs of both referring physicians and lead extractors in the United States. Although our survey from the ACC received responses from a slightly broader range of physicians (386 participants vs 336 EHRA survey participants), it mirrors the EHRA survey’s overall results, highlighting similar deficiencies in guideline adherence and discrepancies between real-world management and guideline recommendations. Both surveys reported a high perceived risk of extraction, potentially hindering patient referrals for lead extraction. These findings collectively underscore a global educational need to enhance health care providers' knowledge and skills to improve patient outcomes in managing CIED infections.

CIED infection remains a major cause of morbidity and mortality in contemporary EP practice. Early identification and optimal management of CIED infection is pivotal in improving short- and long-term patient outcomes. Low levels of familiarity with clinical guidelines for CIED infection management and delayed referral to lead extraction has significant impact on clinical outcomes. This was evident in evaluating the patient case scenarios in the current survey. In a scenario of a pocket infection with methicillin-resistant Staphylococcus aureus bacteremia, only 65% of non-EPs and 33% of PCPs recommended complete system extraction. Even in the setting of complete device erosion, only 51% of non-EPs and 32% of PCPs recommended system extraction. Consideration for extraction was significantly higher for EPs in these scenarios. Current Heart Rhythm Society guidelines provide a Class I indication for complete system extraction in these scenarios.^7^ However, a great divide exists between recommendations and current clinical practice patterns.^10^ The surveyed clinical cases highlight a critical low level of awareness with clinical practice guidelines for management of CIED infections among non-EP cardiologists and PCPs, who are often the first point of contact for patients with device-related issues. In addition, several non-EP cardiologists in community practice implant, follow, and manage CIEDs without an involvement of an EP. The results of the current survey call for increased societal efforts to improve awareness regarding CIED infection management among non-EP cardiologists and PCPs, which in effect will lead to improved patient outcomes.

Timing of lead extraction is crucial in the management of CIED infection. Several prior studies have demonstrated that early identification and system extraction is associated with lower rates of mortality and earlier clearance of systemic infection. Lin et al^12^ reported that delayed CIED extraction in bacteremic patients is associated with an increased risk of septic shock, renal failure, respiratory failure, and decompensated heart failure. In a recent analysis of the National Readmissions Database, Lee et al^13^ evaluated outcomes of early vs delayed CIED extraction. In this study of 12,999 patients undergoing transvenous lead extraction, 68% underwent early (<7 days) extraction while 32% underwent delayed (>7 days) system removal. Delayed lead extraction was associated with higher rates of in-hospital mortality, procedure-related adverse events as well as longer length of stay. In a propensity score-matched analysis, delayed lead extraction was associated with higher in-hospital mortality (OR: 1.47; 95% CI: 1.22-1.76, *P* < 0.001). These studies clearly highlight the need for early referral for lead extraction. In the current survey, approximately 40% of EPs who do not perform lead extraction and 11% of PCPs reported managing CIED infections themselves without a referral to a lead extraction specialist. This is an important cause of delay in receiving appropriate care for patients with CIED infection and represents an opportunity for improved education and raising clinical awareness.

One of the major determinants of referral for lead extraction is perceived risk of the procedure in 86% of the survey respondents. Additionally, one-fourth of PCPs and one-fourth of non-EP cardiologists perceived lead extraction as a procedure with high (6%-10%) or very high (>10%) risk of major complications. While expertise in risk-stratification for device extraction remains critical to avoid catastrophic complications, increasing experience, refinement of techniques, and advances in technology have reduced the risk of complications dramatically.^14^, ^15^, ^16^ Deshmukh et al^17^ evaluated national trends in complication rates of transvenous lead extractions using the Nationwide Inpatient Sample. In this analysis of 91,890 procedures, rate of major vascular complications needing urgent open-heart surgery was 0.2% and rate of in-hospital mortality was 2%. These estimates of complication rates are similar to other electrophysiological procedures such as left atrial appendage occlusion and atrial fibrillation ablation. In a study of 2,319 patients undergoing transvenous lead extraction, rate of mortality within 30 days after the procedure was 3%, with 90% of deaths not related to the extraction procedure.^18^ Predominant cause of death was sepsis and decompensated heart failure. These studies highlight the most adverse outcomes following lead extraction procedures are secondary to comorbidities and not directly related to the procedure itself. Adverse outcomes related to these comorbid conditions are higher with delayed extraction referrals. The current survey shows a clear discrepancy between actual and perceived complication rates of lead extraction, which in turn is the major determinant for referral for an extraction. This divide can be narrowed with efforts to improve education and awareness regarding indications as well as actual complication rates of lead extractions in contemporary clinical practice.

### Study Limitations

This survey depended on voluntary participation and was self-reported with an overall response rate of 20%. The self-reported nature of the responses could perhaps introduce bias, and the relatively low response rate may limit the generalizability of the findings. It is worth noting, however, that physician surveys typically are limited by low response rates, often around 10%. Another potential limitation is the presence of selection bias. The respondents who opted to participate could be those who have a specific clinical interest in CIED infection. Demographics data also showed that a significant number of respondents were from urban/suburban locations. Consequently, they might have a greater familiarity with the practice guidelines compared to the community at large. If this is the case, the actual level of guideline awareness among the broader professional community may be much lower than what the survey results suggest. In our survey, hospital information was not tracked. However, as a national survey with participants from a wide variety of practice settings, the likelihood of multiple respondents coming from the same hospital is relatively low. Moreover, the size of the institution was not included in the survey, preventing us from considering any institution volume factors. Additionally, due to the nature of the survey, the 3 patient case scenarios were simplified and may not accurately reflect real-world clinical decision-making. The scenarios did not consider many other factors that can influence the decision to perform CIED system extraction due to infection. Lastly, this is a cross-sectional survey with no follow-up data; therefore, practice trends cannot be determined.

## Conclusions

In the largest societal-based survey exploring practice patterns in management of CEID infections among 387 physicians, we found low rates of familiarity with guidelines for CIED infection necessitating device extraction among non-EP cardiologists and PCPs. Furthermore, there is a perceived high complication risk and reported lack of management pathways for CIED infections at most institutions. Addressing these gaps, with a focus on education toward general cardiologists and PCPs, may be a key factor in improving outcomes in patients with CIED infections.

PERSPECTIVES **COMPETENCY IN MEDICAL KNOWLEDGE 1:** The findings of this study call for a robust education at all levels of clinical care pathways in CIED infection diagnosis, risk stratification, and management.

**COMPETENCY IN MEDICAL KNOWLEDGE 2:** Often the guidelines and recommendations from subspecialty professional societies do not percolate the nonspecialty or primary care physician education. Improved education and therapy awareness will minimize the disease burden, psychosocial, economic and morbidity and mortality burden imposed by CIED infections across the world.

**COMPETENCY IN PATIENT CARE:** Automated screening processes and directed care through prompts in the electronic medical record systems could be a positive step in the right direction.

## Funding support and author disclosures

This work was supported by Philips Image-Guided Therapy Corporation. The content has not been influenced in any way by its sponsor. Dr Lakkireddy is a consultant for Philips and Abbott and has received honoraria from Abbott, Medtronic, Boston Scientific, and Biosense Webster. Dr Rao has received honoraria from Medtronic, Boston Scientific, and Phillips. Dr Theriot is an employee of American College of Cardiology. Dr Ram is an employee of Philips Image-Guided Therapy Corporation. Dr Gao is an employee of Philips Image-Guided Therapy Corporation. Dr Cheung has received honoraria/consulting fees from Abbott, Biotronik, and Boston Scientific; and has received research support from Boston Scientific and fellowship grant support from Abbott, Biotronik, Boston Scientific, and Medtronic. Dr Birgersdotter-Green has received honoraria from Medtronic, Boston Scientific, Abbott, and Biotronik. All other authors have reported that they have no relationships relevant to the contents of this paper to disclose.

## References

[1] Dai M., Cai C., Vaibhav V. (2019). Trends of cardiovascular implantable electronic device infection in 3 decades. J Am Coll Cardiol EP.

[2] Voigt A., Shalaby A., Saba S. (2006). Rising rates of cardiac rhythm management device infections in the United States: 1996 through 2003. J Am Coll Cardiol.

[3] Wilkoff B.L., Boriani G., Mittal S. (2020). Impact of cardiac implantable electronic device infection: a clinical and economic analysis of the WRAP-IT trial. Circ Arrhythm Electrophysiol.

[4] Sridhar A.R.M., Lavu M., Yarlagadda V. (2017). Cardiac implantable electronic device-related infection and extraction trends in the U.S.: lead infection and extraction trends in the U.S. Pacing Clin Electrophysiol.

[5] Sandoe J.A.T., Barlow G., Chambers J.B. (2015). Guidelines for the diagnosis, prevention and management of implantable cardiac electronic device infection. Report of a joint working party project on behalf of the British Society for Antimicrobial Chemotherapy (BSAC, host organization), British Heart Rhythm Society (BHRS), British Cardiovascular Society (BCS), British Heart Valve Society (BHVS) and British Society for Echocardiography (BSE). J Antimicrob Chemother.

[6] Tarakji K.G., Chan E.J., Cantillon D.J. (2010). Cardiac implantable electronic device infections: presentation, management, and patient outcomes. Heart Rhythm.

[7] Kusumoto F.M., Schoenfeld M.H., Wilkoff B.L. (2017). 2017 HRS expert consensus statement on cardiovascular implantable electronic device lead management and extraction. Heart Rhythm.

[8] Blomström-Lundqvist C., Traykov V., Erba P.A. (2020). European Heart Rhythm Association (EHRA) International Consensus Document on how to prevent, diagnose, and treat cardiac implantable electronic device infections—endorsed by the Heart Rhythm Society (HRS), the Asia Pacific Heart Rhythm Society (APHRS), the Latin American Heart Rhythm Society (LAHRS), International Society for Cardiovascular Infectious Diseases (ISCVID) and the European Society of Clinical Microbiology and Infectious DISEASES (ESCMID) in collaboration with the European association for Cardio-Thoracic Surgery (EACTS). Europace.

[9] Pokorney S. (April 2022). Late Breaking Clinical Trials presented at: American College of Cardiology (ACC).

[10] Sciria C.T., Kogan E.V., Mandler A.G. (2023). Low utilization of lead extraction among patients with infective endocarditis and implanted cardiac electronic devices. J Am Coll Cardiol.

[11] Rao A., Garner D., Starck C. (2020). Knowledge gaps, lack of confidence, and system barriers to guideline implementation among European physicians managing patients with CIED lead or infection complications: a European Heart Rhythm Association/European Society of Cardiology Educational needs assessment survey. Europace.

[12] Lin A.Y., Saul T., Aldaas O.M. (2021). Early vs delayed lead extraction in patients with infected cardiovascular implantable electronic devices. J Am Coll Cardiol EP.

[13] Lee J.Z., Majmundar M., Kumar A. (2022). Impact of timing of transvenous lead removal on outcomes in infected cardiac implantable electronic devices. Heart Rhythm.

[14] Azarrafiy R., Tsang D.C., Wilkoff B.L., Carrillo R.G. (2019). Endovascular occlusion balloon for treatment of superior vena cava tears during transvenous lead extraction: a multiyear analysis and an update to best practice protocol. Circ Arrhythm Electrophysiol.

[15] Darden D., Boateng B.A., Tseng A.S. (2022). Transvenous laser lead extraction in patients with congenital complete heart block. Heart Rhythm.

[16] Brunner M.P., Cronin E.M., Wazni O. (2014). Outcomes of patients requiring emergent surgical or endovascular intervention for catastrophic complications during transvenous lead extraction. Heart Rhythm.

[17] Deshmukh A., Patel N., Noseworthy P.A. (2015). Trends in use and adverse outcomes associated with transvenous lead removal in the United States. Circulation.

[18] Lee J.Z., Tan M.C., Karikalan S. (2022). Causes of early mortality after transvenous lead removal. J Am Coll Cardiol EP.

