# Supplementary material for: Contemporary Management of Cardiac Implantable Electronic Device Infection: The American College of Cardiology COGNITO Survey
Source: JACC Adv. 2023 Dec 20;3(2):100773. doi: 10.1016/j.jacadv.2023.100773 (PMC11198053; doi:10.1016/j.jacadv.2023.100773)
Supplement: Supplementary data [file mmc1.docx]

**2022-02 February 2022 CardioSurve Survey**

Welcome to the CardioSurve February 2022 Survey!

On behalf of the American College of Cardiology, thank you again for your participation in CardioSurve. This survey aims to understand and assess the current practice related to diagnosis and management of CIED infections.

Infection related to Cardiac implantable electronic devices (CIEDs, including pacemakers, defibrillators, etc.) is a life-threatening complication that is increasing in frequency. Comprehensive guidelines for appropriate management exist. However, the clinical presentation may be non-specific and variable, resulting in delayed and sub optimal management with poor outcomes.

When you complete all of the questions be sure to select the "Submit" button.

If you need to exit the survey before you are finished, please select the "Save" button. Your responses will be saved and you can resume taking the survey from the point at which you stopped.

Thank you very much for your time and insights.

To begin...

**Section I: COGNITO Survey on Cardiac Implantable Electronic Devices (CIEDs)**

**DEMOGRAPHIC SECTION**

1. Do you currently treat patients in your practice? (answer required)
2. Yes
3. No **(THANK AND TERMINATE)**
4. Please select your specialty from the following list below. Please select all that apply. (answer required)
5. Cardiologist (General)
6. Interventional Cardiologist
7. Heart Failure Specialist
8. Cardiac Surgeon
9. Electrophysiologist
10. Infectious disease
11. Internist
12. Hospitalist
13. Primary care physician
14. Other, please specify: _________

**[PROGRAMMER NOTE: Q.C THROUGH Q.H ASKED OF OVERSAMPLE AUDIENCES ONLY]**

**[PROGRAMMER NOTE: Do not ask Q.C of “Primary Care Physician” Respondents in Q.B]**

1. How many years have you been in practice after your fellowship training?
   1. Less than 1 year
   2. 1 – 7 years
   3. 8 – 14 years
   4. 15 - 21 years
   5. 22 or more years

**[PROGRAMMER NOTE: Ask Q.CA of “Primary Care Physician” Only Respondents in Q.B]**

CA. How many years have you been in practice after your residency?

- 1. Less than 1 year
  2. 1 – 7 years
  3. 8 – 14 years
  4. 15 - 21 years
  5. 22 or more years

1. What is your gender?
   1. Male
   2. Female
   3. Other, please specify: _______________
   4. Decline to provide
2. Which of the following best describes your primary work setting?
   1. Cardiovascular Group
   2. Government Hospital or Agency-Military
   3. Government Hospital or Agency-Veterans Affairs
   4. Government Hospital or Agency-Other
   5. Insurance Company (HMO, PPO, IPA)
   6. Industry (pharma, device)
   7. Multi-Specialty Group
   8. Medical School/University
   9. Non-governmental Hospital
   10. Solo practice
   11. Physician group
   12. Other, please specify: ____________________
3. To your best estimate, how many full-time cardiologists (40+ hours per week) are in the entire medical practice (i.e.: across all offices)?
   1. None
   2. 1 – 4
   3. 5 – 10
   4. 11 – 25
   5. 26 or more
   6. Not sure
4. What is the location of your practice?
   1. Rural
   2. Suburban
   3. Urban
   4. Decline to answer
5. In which US state or territory is your practice primarily located?

US State or Territory: ____________________

- Do not live in a US state or territory **(THANK AND TERMINATE)**

**MAIN SURVEY SECTION**

1. Do you implant cardiac implantable electronic devices (CIED; pacemaker, defibrillator etc.)?
   1. Yes
   2. No
2. Do you perform lead extraction?
   1. Yes
   2. No
3. Approximately how many years of experience do you have in managing patients with CIED?
   1. None, no experience
   2. Less than 1 year
   3. 1 to 7 years
   4. 8 to 14 years
   5. 15 to 21 years
   6. More than 22 years
4. What is your estimate of an annual CIED infection rate at your center as quoted to patients?

Less than 1%

1% - 5%

> 5%

Not sure

Not applicable

1. In your opinion, which of the following scenarios would you typically associate with a higher rate of CIED infection? Please select all of the apply.
   1. De novo implant
   2. Generator change
   3. Upgrade
   4. Generator change and Upgrade
   5. None of the above

For this next set of questions, we would like to have your perspective regarding the following patient case scenarios.

1. Case 1

63 y male patient with CIED implanted 8 years ago and device replaced 1 year ago. He now presents with recurrent fever.

6A. How would you evaluate this patient? Please select all that apply.

1. Chest X-ray
2. Blood Cultures
3. Implant site check
4. Echocardiogram
5. Other, please specify: ____________________

6B. In this patient, the implant site shows mild redness, and his blood culture came back positive with Methicillin-resistant Staphylococcus aureus. In your opinion, what would be the most appropriate therapy for this patient? Please select only one response.

1. Antibiotic treatment for 4 weeks
2. Explore the pocket, do capsulectomy and wrap generator in antibiotic pouch, followed by 4 weeks of antibiotics
3. Explore the pocket, remove the generator, cap the leads, continue 4 weeks of antibiotics
4. Complete CIED system removal, and 4 weeks of antibiotics
5. Other, please specify: ____________________
6. Case 2

Patient with a pacemaker implanted 7 years ago, presents with fever and a pacemaker implant site shown below. What would your next step typically be in this scenario? Please select only one response.


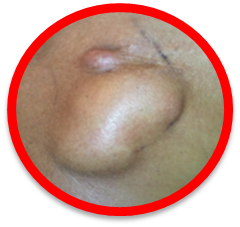


1. Reassure and reassess in 4 weeks
2. Antibiotic treatment alone
3. Open the pocket and clean it out, leaving the pacemaker in situ
4. Antibiotic treatment, remove the generator, cap the leads
5. Antibiotic treatment, complete CIED system removal
6. Other, please specify: ____________________
7. Case 3

Patient with a pacemaker implanted 7 years ago presents for routine physical examination with 97.3°F, HR 60bpm, BP: 120/80 mmHg, WBC 7.8 x 10^3^/μL. The implant site is shown below. For this scenario, what would you typically do next? Please select only one response.


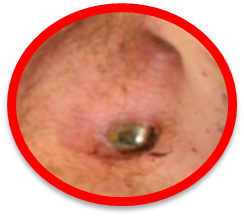


1. Antibiotic treatment and reassessment in 6 weeks
2. Explore the pocket, excise the affected area, reposition the generator with antibiotic pouch wrapped, followed by 6 weeks of antibiotics
3. Generator removal, cut the lead to retract into venous system, and continue antibiotics for 6 weeks
4. Complete CIED system removal, and 6 weeks of antibiotics
5. Other, please specify: ____________________
6. How familiar are you with the current practice guidelines (HRS/AHA/EHRA) recommendations for CIED infection extraction/removal?
7. Not at all familiar
8. Not very familiar
9. Somewhat familiar
10. Very familiar
11. Do not know
12. Does your institution/department have practice guidelines based protocols in place for managing patients with CIED infection?
13. Yes
14. No
15. Do not know
16. When you diagnose a patient with CIED pocket infection, do you typically... Please select all that apply.
    1. Manage the patient on your own
    2. Refer the patient to device specialist who has expertise in CIED management
    3. Refer the patient to an infectious disease specialist, and plan therapy based on their recommendation
    4. Other, please specify: ____________________
17. When you recognize bacteremia in a patient with CIED, do you generally... Please select all that apply.
    1. Manage the patient on your own
    2. Refer the patient to device specialist who has expertise in CIED management
    3. Refer the patient to an infectious disease specialist, and plan therapy based on their recommendation
    4. Other, please specify: ____________________
18. How often do you consult an infectious disease specialist to assist in the management of CIED infection patients?
19. Always
20. Often
21. Sometimes
22. Rarely
23. Never
24. In your opinion, what is the risk level of major complications (e.g., vascular complications, mortality, etc.) resulting from a lead extraction procedure?
    1. Very high risk, >10%
    2. High risk, 6 - 10%
    3. Medium risk, 1 - 5%
    4. Low risk, <1%
    5. Not sure
25. In general, when considering lead extraction for a patient, to what degree do each of the following influence your decision to consider extraction (or referral for extraction)?

|  | No influence at all | Somewhat of an influence | Strong influence | Not sure |
| --- | --- | --- | --- | --- |
| Age of the lead |  |  |  |  |
| Patient’s co morbidity |  |  |  |  |
| Age of patient |  |  |  |  |
| Ease of access to extraction centre |  |  |  |  |
| Fear of losing the patient to the extraction physician if I refer them on |  |  |  |  |
| Risk of lead extraction procedure |  |  |  |  |

1. The ACC is considering the development of education focused on issues related to CIED. How likely would you be to participate in this type of education?

| Not at all likely - 1 | 2 | 3 | 4 | Extremely likely - 5 | Not sure |
| --- | --- | --- | --- | --- | --- |
|  |  |  |  |  |  |

1. In terms of formats for this type of clinician education, which of the following formats would you prefer? Please select all that apply.
   1. Podcast series
   2. Interactive info-graphic focused on guidelines
   3. Online education/e-learning
   4. Expert written analysis
   5. Case studies
   6. Other, please specify: ___________________
   7. Not sure

Q.LAST Please feel free to comment about any of the topics presented in this survey.

___________________________________________________________________ ___

___________________________________________________________________ ___

The results of this survey will be collected and presented by its authors. All information will be kept confidential and anonymous. Thank you for completing this survey!

Please click "SUBMIT" to record your responses.
